# Liver Status Assessment After Coronary Artery Bypass Grafting

**DOI:** 10.7759/cureus.72210

**Published:** 2024-10-23

**Authors:** Andreea Ludusanu, Bogdan M Ciuntu, Adelina Tanevski, Marin Fotache, Viorel D Radu, Alexandru Burlacu, Grigore Tinica

**Affiliations:** 1 Anatomy, Grigore T. Popa University of Medicine and Pharmacy, Iași, ROU; 2 General Surgery, Faculty of Medicine, Grigore T. Popa University of Medicine and Pharmacy, Iași, ROU; 3 Faculty of Economics and Business Administration, Alexandru Ioan Cuza University, Iași, ROU; 4 Urology, Grigore T. Popa University of Medicine and Pharmacy, Iași, ROU; 5 Cardiology, Institute of Cardiovascular Diseases "Prof. Dr. George I. M. Georgescu", Iași, ROU; 6 Cardiac Surgery, Faculty of Medicine, Grigore T. Popa University of Medicine and Pharmacy, Iași, ROU; 7 Cardiac Surgery, Faculty of Medicine, Institute of Cardiovascular Diseases "Prof. Dr. George I. M. Georgescu", Iași, ROU

**Keywords:** cad: coronary artery disease, coronary artery bypass grafting(cabg), european system for cardiac operative risk evaluation, hospital outcomes, liver dysfunctions, model for end stage liver disease (meld)

## Abstract

Background

Coronary artery bypass grafting (CABG) is a common surgical intervention used to treat severe coronary artery disease. The Model for End-Stage Liver Disease (MELD) score has become a widely used prognostic index for assessing the severity of liver disease and prioritizing liver transplantation. However, its utility in predicting outcomes in cardiac surgery procedures has not been extensively evaluated.

Methods

This retrospective study gathered data on patients who underwent CABG or CABG combined with other concomitant surgical interventions, such as carotid common or external carotid artery endarterectomy, thoracic aortic aneurysm repair, and aortic or mitral valve replacement or decalcification procedures, at a single tertiary care facility from January 2011 to December 2020. Researchers collected demographic, clinical, and laboratory information, including MELD score and European System for Cardiac Operative Risk Evaluation (EuroSCORE) data. The patients were divided into two groups: the first group included only those who underwent CABG, while the second group comprised patients who underwent CABG along with other concomitant cardiac interventions.

Results

The MELD score at discharge was significantly higher in the CABG and other interventions group compared to the CABG-only group (median = 14.09, IQR = 7.41-18.7 vs. median = 6.41, IQR = 4.61-9.44, p < 0.001). However, the difference in MELD score at admission between the two groups was not statistically significant (p = 0.328). A p-value < 0.05 was considered statistically relevant, indicating that liver function worsened postoperatively in the patients with additional interventions. The EuroSCORE was also significantly higher in the CABG and other interventions group, suggesting a higher surgical risk as expected (median = 5.74, IQR = 3.54-11.47 vs. median = 3.34, IQR = 1.97-5.66, p < 0.001). Additionally, differences in laboratory parameters, especially coagulation and hemostasis indicators throughout the postoperative period, including the ICU stay (divided into four equal periods based on each patient’s total ICU length of stay) and at discharge, indicate a more complex biological state in patients with additional interventions. These findings may have implications for perioperative management and long-term outcomes.

Conclusions

The elevated MELD score in patients undergoing CABG with additional interventions emphasizes the need for close monitoring of liver function and coagulation status. Evaluating hepatic status preoperatively would be beneficial, and incorporating liver-protective strategies could help mitigate postoperative repercussions. It may also be useful to include liver function parameters in existing cardiovascular risk scores to improve risk assessment.

## Introduction

With an aging population and advancements in cardiac surgery, more patients with liver dysfunction are undergoing heart procedures [1-3]. Liver dysfunction in these patients may arise from the severity of heart damage, manifesting as cardiac hepatopathy, also known as congestive hepatopathy or heart cirrhosis, or from other hepatic conditions, such as viral hepatitis, amiodarone toxicity, and non-alcoholic fatty liver disease [4-6]. Patients with preoperative liver dysfunction face higher risks of both pre- and postoperative complications compared to those with healthy livers [7], and they also have higher mortality risks following cardiac surgery [8]. Therefore, accurately assessing the severity of liver dysfunction before surgery is crucial for risk stratification in cardiac patients [8]. While studies have explored postoperative mortality in cardiac surgery patients with cirrhosis, liver dysfunction has not yet been integrated into existing cardiovascular surgery risk models [8]. Various liver function tests have been suggested as potential predictors of surgical risk in patients with liver disease. Historically, the Child-Turcotte-Pugh (CTP) classification was used; however, recent studies suggest that the Model for End-Stage Liver Disease (MELD) score provides a more reliable and objective measure [3,4,7-10]. Initially developed to predict mortality in transjugular intrahepatic portosystemic shunt (TIPS) procedures, the MELD score is now widely used to assess liver dysfunction severity and prioritize liver transplants [11]. Additionally, numerous studies have validated the MELD score’s effectiveness in predicting short-term survival in acute liver failure, acute alcoholic hepatitis, and cirrhosis patients undergoing non-transplant surgeries [11,12]. Moreover, the MELD score and its variants have been shown to accurately estimate mortality risk in cases of acute heart failure, regardless of the underlying cause [13,14]. Prior research on the prognostic significance of the CTP classification and MELD score in cardiac surgery has been limited by small sample sizes and definitive cirrhosis diagnoses [15,16].

The risk of coronary artery disease (CAD) increases with age, affecting 49% of men and 32% of women over 40 years of age [17]. While women generally experience lower incidence rates until about a decade later, their risk increases significantly after menopause [18]. Despite advances in treatment, surgical interventions such as percutaneous coronary intervention (PCI) and coronary artery bypass grafting (CABG) remain essential long-term treatments for severe CAD. CABG is a widely performed procedure, with over 200,000 surgeries conducted annually in the United States, aimed at improving blood supply to the heart in patients with advanced CAD [19]. While the surgery is generally well-tolerated, non-cardiac complications, including those related to liver dysfunction, can occur [20].

Assessing a patient’s liver function using the MELD score before surgery can offer valuable insights into their perioperative risk profile [21,22]. Many CABG patients have additional medical conditions that increase their risk of perioperative complications and mortality, including advanced age, impaired renal function, heart failure, and prior strokes [23,24]. This retrospective analysis aimed to investigate the relationship between liver dysfunction, as assessed by the MELD score, and postoperative outcomes in patients undergoing coronary artery bypass surgery. This included patients who underwent CABG alone or in combination with other interventions, such as aortic and mitral valve replacement, valve decalcification procedures, carotid endarterectomy, repair of thoracic aortic aneurysms, and other concurrent surgeries. Secondly, we evaluated the intra- and postoperative evolution of these patients from both biological and echocardiographic perspectives.

## Materials and methods

A cohort of 699 adult patients underwent CABG at the “Prof. Dr. George I.M. Georgescu” Institute of Cardiovascular Diseases of Iași, from January 1, 2011, to December 31, 2020. The procedures involved median sternotomy and the use of a heart-lung machine. The inclusion criteria for patients were as follows: CABG patients with all biological data needed to calculate the MELD score, including bilirubin (mg/dL), creatinine (mg/dL), and the International Normalized Ratio (INR) upon admission and discharge, along with complete intraoperative protocol information such as aorta clamping time (min), total extracorporeal circulation time (min), height (cm), and weight (kg). Additional data collected included general patient information such as age (years), sex, Rh factor, blood type, and length of hospitalization (days). We analyzed variables to establish the biological status of the patients, including hemoglobin (g/dL), hematocrit (%), leukocytes (×10³/μL), neutrophils (%), lymphocytes (×10³/μL), monocytes (%), eosinophils (×10³/μL), platelets (×10³/μL), erythrocyte sedimentation rate (mm/h), activated partial thromboplastin time (APTT, sec), prothrombin time (PT, sec), prothrombin ratio (%), fibrinogen (mg/dL), sodium (mEq/L), potassium (mEq/L), blood glucose (mg/dL), urea (mg/dL), triglycerides (mg/dL), aspartate aminotransferase (GOT, U/L), alanine aminotransferase (GPT, U/L), lactate dehydrogenase (LDH, U/L), and cholesterol (mg/dL). Imaging data were also collected, including electrocardiogram findings (RS segment, PR segment, QRS complex, QT segment, TS segment, and QRS axis), the cardiothoracic ratio (CTR) from radiological examination, and echocardiography results such as the end-diastolic diameter of the left ventricle (mm), the end-systolic diameter of the left ventricle (mm), fractional shortening (%), ejection fraction (%), volumetric ejection fraction (%), mitral valve orifice area (cm²), a maximum gradient across the tricuspid valve (dPmax, mmHg), tricuspid annular plane systolic excursion (TAPSE, mm), and the degree of mitral, aortic, pulmonary, and tricuspid valve insufficiency.

Exclusion criteria included patients with incomplete data for MELD score calculation upon admission and discharge and those with incomplete postoperative follow-up forms. To ensure anonymity, each patient was assigned a code number, and the study findings were utilized in accordance with a protocol sanctioned by the Research Ethics Committee of Grigore T. Popa University of Medicine and Pharmacy, Iași (EC No. 163, March 21, 2022). After extracting all relevant data, the patients were divided into two groups: those who underwent only CABG surgery and those who underwent CABG combined with other cardiac interventions, such as aortic and mitral valve replacements, valve decalcification procedures, carotid endarterectomy, thoracic aortic aneurysm repair, and other concomitant surgical procedures.

We calculated the MELD score using the patient's admission and discharge bilirubin, creatinine, and INR, according to the official formula: MELD score = 10 × ((0.957× ln(Creatinine)) + (0.378 × ln(Bilirubin)) + (1.12 × ln(INR))) + 6.43 [24]. We also calculated the EuroSCORE on admission using the official calculator on the EuroScore website [25]. Patients were stratified into three groups based on their preoperative MELD score: low-risk (MELD: <10), intermediate-risk (MELD: 10-19), and high-risk (MELD: ≥20). Low-risk included individuals with an MELD score less than 10, intermediate-risk included those with an MELD score between 10 and 19, and high-risk included those with an MELD score of 20 or greater [24].

The total days of stay in the intensive care unit (ICU) were divided into four equal periods based on each patient’s length of stay to assess their evolution during the immediate postoperative period. The resulting ICU balance sheet consisted of four stages: (1) INITIAL (from admission to the ICU), (2) 2/3 of the stay (two-thirds into the ICU stay), (3) 3/3 of the stay (at the end of the ICU stay), and (4) FINAL (at discharge from the ICU). At each stage, the following parameters were recorded: urea, creatinine, creatine kinase-MB, hemoglobin, hematocrit, leukocytes, thrombocytes, red blood cells, prothrombin time, total bilirubin, aspartate aminotransferase, alanine aminotransferase, prothrombin activity (sec), INR, prothrombin ratio (%), fibrinogen, and APTT.

The study data were analyzed using IBM SPSS Statistics for Windows, Version 25 (Released 2017; IBM Corp., Armonk, New York) and visualized using Microsoft Office Excel/Word 2021 (Microsoft Corporation, Redmond, Washington). Quantitative variables were assessed for normal distribution through the Shapiro-Wilk test and reported as either means with standard deviations or medians with interquartile ranges, as appropriate. Non-parametric variables were compared between groups using the Mann-Whitney U test. Qualitative variables were summarized as counts or percentages and compared between groups using Fisher’s exact test. A significance level of α = 0.05 was applied to all statistical tests, with p-values < 0.05 considered statistically significant [26].

## Results

Of the 699 patients who underwent CABG over the 10-year period, 110 were excluded based on the predefined exclusion criteria, resulting in 589 patients included in the study. Of these, 426 were assigned to Group 1 (CABG only), and the remaining 163 were assigned to Group 2 (CABG with other surgical interventions). A comparison of demographic characteristics between the two groups showed that patients in Group 2 had higher age (median = 67, IQR = 60-72 vs. median = 63, IQR = 57-68, p < 0.001), a higher proportion of women (28.8% vs. 18.5%, p = 0.010), and longer hospitalization periods (median = 19, IQR = 15-24 vs. median = 17, IQR = 14-22, p < 0.001). Additionally, Group 2 patients had higher MELD Scores at discharge (median = 14.09, IQR = 7.41-18.7 vs. median = 6.41, IQR = 4.61-9.44, p < 0.001) and higher EUROScores (median = 5.74, IQR = 3.54-11.47 vs. median = 3.34, IQR = 1.97-5.66, p < 0.001) compared to Group 1. However, there was no significant difference in MELD scores at admission between the two groups (p = 0.328), as shown in Table 1.

**Table 1:** Comparison of demographic characteristics between analyzed groups *p<0.05. IQR: interquartile range, MELD: model for end-stage liver disease, EuroSCORE: European System for Cardiac Operative Risk Evaluation.

Group/Parameter	Bypass, Median (IQR) (N=426)	Bypass and Other Interventions, Median (IQR) (N=163)	P-value*
Age (years)	63 (57-68)	67 (60-72)	<0.001
Gender (male) (number)	347	116	0.010
Hospitalization period (days)	17 (14-22)	19 (15-24)	<0.001
MELD score - Admission	5.5 (3.43-8.16)	6.04 (3.74-8.5)	0.328
MELD score - Discharge	6.41 (4.61-9.44)	14.09 (7.41-18.7)	<0.001
EuroSCORE	3.34 (1.97-5.66)	5.74 (3.54-11.47)	<0.001

The analysis of laboratory parameters at admission between the two groups shows that patients with bypasses and other interventions had lower concentrations of hemoglobin (median = 13.52, IQR = 12.6-14.5 vs. median = 13.9, IQR = 12.9-14.7, p = 0.016), hematocrit (median = 40.1, IQR = 37.7-42.8 vs. median = 41.1, IQR = 38.34-43.4, p = 0.011), lymphocyte percentage (median = 24.7, IQR = 20.1-30.75 vs. median = 27.15, IQR = 22-32.7, p = 0.006), GPT (median = 28, IQR = 20-41 vs. median = 30, IQR = 22-50, p = 0.034), and triglycerides (median = 113.5, IQR = 87-160 vs. median = 125.5, IQR = 93.5-178, p = 0.038) compared to patients with only bypasses. In contrast, these patients exhibited higher neutrophil percentages (median = 64.3, IQR = 57.5-70.35 vs. median = 62.4, IQR = 55.8-68.17, p = 0.011) and total cholesterol concentrations (median = 167, IQR = 139.5-200 vs. median = 157, IQR = 133-189, p = 0.020), as shown in Table 2. From an echocardiographic perspective at admission, patients with bypasses and other interventions also had higher values for the CTR (median = 0.49, IQR = 0.46-0.53 vs. median = 0.46, IQR = 0.43-0.5, p < 0.001) and dPmax (median = 30.5, IQR = 23-38 vs. median = 22, IQR = 17-30, p < 0.001). However, these patients had lower EF% values (median = 45, IQR = 35-53.25 vs. median = 50, IQR = 43-60, p < 0.001) and TAPSE values (median = 22, IQR = 19.5-25 vs. median = 24, IQR = 22-26, p = 0.003) compared to those with only bypasses. All these data are summarized in Table 3.

**Table 2:** Comparison of laboratory parameters measured at admission between analyzed groups *p<0.05. IQR: interquartile range, GPT: glutamate pyruvate transaminase.

Group/Parameter	Bypass, Median (IQR) (N=426)	Bypass and Other Interventions, Median (IQR) (N=163)	P-value*
Hemoglobin (g/dL)	13.9 (12.9-14.7)	13.52 (12.6-14.5)	0.016
Hematocrit (%)	41.1 (38.34-43.4)	40.1 (37.7-42.8)	0.011
Neutrophils (%)	62.4 (55.8-68.17)	64.3 (57.5-70.35)	0.027
Lymphocytes (%)	27.15 (22-32.7)	24.7 (20.1-30.75)	0.006
GPT (U/L)	30 (22-50)	28 (20-41)	0.034
Triglycerides (mg/dL)	125.5 (93.5-178)	113.5 (87-160)	0.038
Total cholesterol (mg/dL)	157 (133-189)	167 (139.5-200)	0.020

**Table 3:** Comparison of echocardiographic parameters measured at admission between analyzed groups *p<0.05. CTR: cardiothoracic ratio, EF: ejection fraction, dPmax: maximum pressure gradient, TAPSE: tricuspid annular plane systolic excursion.

Group/Parameter	Bypass, Median (IQR) (N=426)	Bypass and Other Interventions, Median (IQR) (N=163)	P-value*
CTR	0.46 (0.43-0.5)	0.49 (0.46-0.53)	<0.001
EF (%)	50 (43-60)	45 (35-53.25)	<0.001
dPmax (mmHg)	22 (17-30)	30.5 (23-38)	<0.001
TAPSE (mm)	24 (22-26)	22 (19.5-25)	0.003

The analysis of laboratory parameters at discharge between the two groups shows a higher percentage of neutrophils (median = 65.8, IQR = 59.1-73.9 vs. median = 64.2, IQR = 57.9-69.62, p = 0.018), total bilirubin (median = 0.78, IQR = 0.61-1.06 vs. median = 0.74, IQR = 0.57-0.98, p = 0.045), APTT (median = 36, IQR = 30.75-47.15 vs. median = 32, IQR = 28.3-35.7, p < 0.001), PT (median = 30.1, IQR = 17.85-36.55 vs. median = 15.8, IQR = 14.8-16.85, p < 0.001), and INR (median = 2.34, IQR = 1.19-2.93 vs. median = 1.12, IQR = 1.05-1.22, p < 0.001), along with a lower percentage of eosinophils (median = 2.4, IQR = 1.7-3.67 vs. median = 2.7, IQR = 1.9-3.9, p = 0.028), triglycerides (median = 146, IQR = 117-182.5 vs. median = 168, IQR = 136.5-216.5, p = 0.007), and PR (median = 31, IQR = 23-62.5 vs. median = 82, IQR = 73-92, p < 0.001) in patients with bypasses and other interventions compared to those with only bypasses, as shown in Table 4. The analysis of echocardiographic parameters at discharge between the groups shows lower EF% values (median = 45, IQR = 35.25-50 vs. median = 50, IQR = 43.5-58, p = 0.001) and TAPSE values (median = 15.5, IQR = 12-18 vs. median = 18, IQR = 15-19, p = 0.013) in patients with bypasses and other interventions compared to patients with only bypasses, as shown in Table 5.

**Table 4:** Comparison of laboratory parameters measured at discharge between analyzed groups *p<0.05. APTT: activated partial thromboplastin time, PT: prothrombin time, PR: prothrombin ratio, INR: international normalized ratio.

Group/Parameter	Bypass, Median (IQR) (N=426)	Bypass and Other Interventions, Median (IQR) (N=163)	P-value*
Neutrophils (%)	64.2 (57.9-69.62)	65.8 (59.1-73.9)	0.018
Eosinophils (%)	2.7 (1.9-3.9)	2.4 (1.7-3.67)	0.028
Total bilirubin (mg/dL)	0.74 (0.57-0.98)	0.78 (0.61-1.06)	0.045
Triglycerides (mg/dL)	168 (136.5-216.5)	146 (117-182.5)	0.007
APTT (sec)	32 (28.3-35.7)	36 (30.75-47.15)	<0.001
PT (sec)	15.8 (14.8-16.85)	30.1(17.85-36.55)	<0.001
PR (%)	82 (73-92)	31 (23-62.5)	<0.001
INR	1.12 (1.05-1.22)	2.34 (1.19-2.93)	<0.001

**Table 5:** Comparison of echocardiographic parameters measured at discharge between analyzed groups *p<0.05. EF: ejection fraction, TAPSE: tricuspid annular plane systolic excursion.

Group/Parameter	Bypass, Median (IQR) (N=426)	Bypass and Other Interventions, Median (IQR) (N=163)	P-value*
EF (%)	50 (43.5-58)	45 (35.25-50)	0.001
TAPSE (mm)	18 (15-19)	15.5 (12-18)	0.013

The comparison of intraoperative parameters between the analyzed groups shows a higher aortic clamping time (median = 97, IQR = 82-119 vs. median = 78, IQR = 63.75-95, p < 0.001), total extracorporeal circulation time (median = 131.5, IQR = 110-159.7 vs. median = 106, IQR = 91.75-126, p < 0.001), and ICU admission time (median = 7, IQR = 5-9 vs. median = 6, IQR = 5-8, p < 0.001) in patients with bypasses and other interventions compared to those with only bypasses. Regarding these parameters, patients with bypasses and other interventions also had lower height (median = 170, IQR = 162-175 vs. median = 170, IQR = 165-175, p = 0.031) and weight (median = 82, IQR = 69-93.25 vs. median = 84, IQR = 76-95, p = 0.027) compared to patients with only bypasses, as shown in Table 6.

**Table 6:** Comparison of intraoperative parameters between analyzed groups *p<0.05. IQR: interquartile range, ICU: intensive care unit.

Group/Parameter	Bypass, Median (IQR) (N=426)	Bypass and Other Interventions, Median (IQR) (N=163)	P-value*
Aortic clamping time (min)	78 (63.75-95)	97 (82-119)	<0.001
Total extracorporeal circulation time (min)	106 (91.75-126)	131.5 (110-159.7)	<0.001
Height (cm)	170 (165-175)	170 (162-175)	0.031
Weight (kg)	84 (76-95)	82 (69-93.25)	0.027
ICU admission time (days)	6 (5-8)	7 (5-9)	<0.001

Differences in laboratory parameters measured during the first period of ICU admission between the analyzed groups show lower values for GPT (median = 24, IQR = 17-36.25 vs. median = 27.5, IQR = 19-46, p = 0.011), hematocrit (median = 29.91, IQR = 27.57-32.22 vs. median = 30.6, IQR = 28.5-32.91, p = 0.049), platelets (median = 111.5, IQR = 92-136.25 vs. median = 132, IQR = 111.2-159, p < 0.001), and PR (median = 55.5, IQR = 49-62 vs. median = 57, IQR = 51-63, p = 0.046), along with higher values for LDH (median = 704.5, IQR = 566-850 vs. median = 627, IQR = 508.5-731, p < 0.001), urea (median = 38, IQR = 31-46 vs. median = 35, IQR = 28-43, p = 0.002), PT (median = 19.2, IQR = 18.1-20.5 vs. median = 18.9, IQR = 17.7-20.1, p = 0.030), and INR (median = 1.46, IQR = 1.37-1.59 vs. median = 1.43, IQR = 1.33-1.55, p = 0.018) in patients with bypasses and other interventions compared to those with only bypasses, as shown in Table 7. In the second period of ICU admission, differences in laboratory parameters between the two groups reveal lower values for GPT (median = 36, IQR = 24.25-63.75 vs. median = 47, IQR = 32-72, p = 0.015) and platelets (median = 130.5, IQR = 105-166.2 vs. median = 151, IQR = 121.5-184.5, p < 0.001), along with higher values for LDH (median = 711, IQR = 558-854.2 vs. median = 574, IQR = 494-747.2, p = 0.002), creatinine (median = 1.21, IQR = 1-1.68 vs. median = 1.08, IQR = 0.92-1.32, p = 0.005), urea (median = 49, IQR = 39-67.5 vs. median = 40, IQR = 30-54, p < 0.001), and APTT (median = 41.5, IQR = 36.1-50.95 vs. median = 38.9, IQR = 35.42-43.95, p = 0.009) in patients with bypasses and other interventions compared to those with only bypasses, as shown in Table 8.

**Table 7:** Comparison of laboratory parameters measured in the first period of ICU admission between analyzed groups *p<0.05. GPT: glutamate pyruvate transaminase, LDH: lactate dehydrogenase, PT: prothrombin time, PR: prothrombin ratio, INR: international normalized ratio.

Group/Parameter	Bypass, Median (IQR) (N=426)	Bypass and Other Interventions, Median (IQR) (N=163)	P-value*
GPT (U/L)	27.5 (19-46)	24 (17-36.25)	0.011
LDH (U/L)	627 (508.5-731)	704.5 (566-850)	<0.001
Urea (mg/dL)	35 (28-43)	38 (31-46)	0.002
Hematocrit (%)	30.6 (28.5-32.91)	29.91 (27.57-32.22)	0.049
Platelets (×10^3 ^/μL)	132 (111.2-159)	111.5 (92-136.25)	<0.001
PT (sec)	18.9 (17.7-20.1)	19.2 (18.1-20.5)	0.030
PR (%)	57 (51-63)	55.5 (49-62)	0.046
INR	1.43 (1.33-1.55)	1.46 (1.37-1.59)	0.018

**Table 8:** Comparison of laboratory parameters measured in the second period of ICU admission between analyzed groups *p<0.05. GPT: glutamate pyruvate transaminase, LDH: lactate dehydrogenase, APTT: activated partial thromboplastin time.

Group/Parameter	Bypass, Median (IQR) (N=426)	Bypass and Other Interventions, Median (IQR) (N=163)	P-value*
GPT (U/L)	47 (32-72)	36 (24.25-63.75)	0.015
LDH (U/L)	574 (494-747.2)	711 (558-854.2)	0.002
Creatinine (mg/dL)	1.08 (0.92-1.32)	1.21 (1-1.68)	0.005
Urea (mg/dL)	40 (30-54)	49 (39-67.5)	<0.001
Platelets (×10^3 ^/μL)	151 (121.5-184.5)	130.5 (105-166.2)	<0.001
APTT (sec)	38.9 (35.42-43.95)	41.5 (36.1-50.95)	0.009

Differences in laboratory parameters measured during the third period of ICU admission between the analyzed groups show higher values for LDH (median = 638, IQR = 587.5-960.5 vs. median = 464.5, IQR = 414.5-542, p = 0.005), WBC (median = 10.3, IQR = 8.1-12.53 vs. median = 9.6, IQR = 7.9-11.9, p = 0.046), PT (median = 17.8, IQR = 15.8-25.55 vs. median = 16.5, IQR = 15.4-17.5, p = 0.001), INR (median = 1.32, IQR = 1.11-2.04 vs. median = 1.17, IQR = 1.1-1.31, p = 0.002), and APTT (median = 41.8, IQR = 35.2-52 vs. median = 36, IQR = 31.9-40.7, p < 0.001). Lower values were observed for fibrinogen (median = 814.8, IQR = 673-942.6 vs. median = 882.2, IQR = 771-968.4, p = 0.014) and PR (median = 65, IQR = 36-84 vs. median = 78, IQR = 65.25-85, p = 0.003) in patients with bypasses and other interventions compared to those with only bypasses, as shown in Table 9. In the fourth period of ICU admission, differences in laboratory parameters between the analyzed groups show higher values for PT (median = 24.25, IQR = 16.1-34.12 vs. median = 16.85, IQR = 15.6-18.1, p = 0.010), INR (median = 1.92, IQR = 1.16-2.89 vs. median = 1.21, IQR = 1.15-1.32, p = 0.011), and APTT (median = 44.4, IQR = 37.95-53.7 vs. median = 36, IQR = 31.07-45.65, p = 0.004), with lower values for PR (median = 40, IQR = 25-79.5 vs. median = 75, IQR = 66.5-81.5, p = 0.007) in patients with bypasses and other interventions compared to those with only bypasses, as shown in Table 10.

**Table 9:** Comparison of laboratory parameters measured in the third period of ICU admission between analyzed groups *p<0.05. LDH: lactate dehydrogenase, WBC: white blood cell, PT: prothrombin time, PR: prothrombin ratio, INR: international normalized ratio, APTT: activated partial thromboplastin time.

Group/Parameter	Bypass, Median (IQR) (N=426)	Bypass and Other Interventions, Median (IQR) (N=163)	P-value*
LDH (U/L)	464.5 (414.5-542)	638 (587.5-960.5)	0.005
WBC (×10^3 ^/μL)	9.6 (7.9-11.9)	10.3 (8.1-12.53)	0.046
Fibrinogen (mg/dL)	882.2 (771-968.4)	814.8 (673-942.6)	0.014
PT (sec)	16.5 (15.4-17.5)	17.8 (15.8-25.55)	0.001
PR (%)	78 (65.25-85)	65 (36-84)	0.003
INR	1.17 (1.1-1.31)	1.32 (1.11-2.04)	0.002
APTT (sec)	36 (31.9-40.7)	41.8 (35.2-52)	<0.001

**Table 10:** Comparison of laboratory parameters measured in the fourth period of ICU admission between analyzed groups *p<0.05. PT: prothrombin time, PR: prothrombin ratio, INR: international normalized ratio, APTT: activated partial thromboplastin time.

Group/Parameter	Bypass, Median (IQR) (N=426)	Bypass and Other Interventions, Median (IQR) (N=163)	P-value*
PT (sec)	16.85 (15.6-18.1)	24.25(16.1-34.12)	0.010
PR (%)	75 (66.5-81.5)	40 (25-79.5)	0.007
INR	1.21 (1.15-1.32)	1.92 (1.16-2.89)	0.011
APTT (sec)	36 (31.07-45.65)	44.4 (37.95-53.7)	0.004

## Discussion

The MELD score can be a powerful tool for assessing postoperative outcomes, as higher scores are associated with an increased risk of complications and mortality following surgical procedures [27]. Current risk stratification methods used in cardiac surgery, such as the STS Short-Term Risk Calculator or EuroSCORE, are limited in evaluating patients with pre-existing liver disease, whether the damage is mild or severe [28]. As noted in this retrospective study, patients who underwent bypass procedures along with other interventions were older, predominantly women, had longer hospital stays, and higher EuroSCORE, suggesting a greater comorbidity burden and heightened mortality risk compared to those who underwent only CABG. The higher MELD score at discharge for the group receiving additional interventions, despite no significant difference in admission MELD scores between the two groups, suggests worsening liver function with increased surgical complexity, leading to worse clinical outcomes. The analysis of laboratory parameters at admission between the two groups revealed a significantly worse coagulation profile and higher cholesterol levels in the group that underwent CABG with additional interventions, compared to the CABG-only group. This group also showed severely impaired cardiac parameters, such as lower EF (%) and TAPSE values, indicating a more severe clinical status at admission and necessitating more intensive medical management [29].

Longer durations of cardiopulmonary bypass are linked to increased morbidity and mortality following cardiac surgery. Impairment of vital organ function, which is crucial for postoperative outcomes, can worsen with prolonged cross-clamp and cardiopulmonary bypass times [30]. This observation aligns with the present study, where patients with additional interventions had longer cross-clamping times, total extracorporeal circulation times, and ICU stays compared to the CABG-only group. Risk assessment tools like the MELD score can help identify patients at higher risk of adverse surgical outcomes, though the lack of randomized controlled trial data introduces uncertainty to this assessment [31]. The postoperative course of patients undergoing various cardiac surgical procedures highlights a complex interplay among inflammatory, coagulation, and fibrinolysis systems [32]. Patients who underwent more extensive surgical interventions, beyond CABG alone, exhibited a heightened inflammatory response and disruptions in coagulation and fibrinolysis. These changes appear to be driven by factors such as blood loss, ischemia-reperfusion injury, and the complexity of the surgical procedure [33,34]. In analyzing the evolution of our patients during the postoperative period, we observed that, during the first and second periods of ICU stay, the group with additional interventions had lower values for GPT, hematocrit, platelets, and PR, along with higher values for LDH, creatinine, and INR, indicating more severe hepatic and renal deterioration. In the third and fourth periods, patients with CABG and additional interventions exhibited higher values for LDH, WBC, PT, INR, and APTT, along with lower fibrinogen and PR values compared to the CABG-only group, reflecting a greater degree of inflammation and coagulation abnormalities [35].

In addition to the modified values in the postoperative period, those related to coagulation and renal function, the bilirubin value at discharge was also significantly higher in patients with CABG and other interventions compared to patients with only CABG, along with a higher percentage of neutrophils, indicating greater postoperative liver dysfunction in the former group. The observed changes in coagulation parameters may be attributed to the complex pathophysiological processes triggered by the combination of bypass procedures and other interventions. Bypass procedures can activate the complement system and increase blood prostacyclin levels, contributing to the accumulation of polymorphonuclear leukocytes in the lungs [36,37]. This pulmonary leukostasis and complement activation may exacerbate coagulation abnormalities. Additionally, the physiological disturbances associated with cardiopulmonary bypass, such as hypoxia, hypothermia, and metabolic acidosis, can further disrupt the coagulation system [37,38].

In summary, the findings from this retrospective study suggest that patients undergoing coronary artery bypass surgery with additional intraoperative interventions exhibit worse laboratory indicators of coagulation and hemostasis in the postoperative period compared to those who underwent only CABG [39,40]. This may contribute to increased rates of adverse clinical outcomes in this higher-risk patient population [41]. The higher MELD score at discharge in patients with more complex procedures supports the hypothesis that these patients experience more severe coagulopathy and end-organ dysfunction during the postoperative period [41,42]. These findings highlight the importance of more intensive monitoring and management of coagulation parameters for patients undergoing CABG and other surgical interventions in the ICU setting [43,44].

The retrospective, single-center design of this study introduces several limitations, such as the possibility of selection bias from the inclusion and exclusion criteria. The use of both the MELD and EuroSCORE for risk adjustment, each including creatinine, introduces some overlap, potentially underestimating the influence of the MELD score on factors that depend on this value [45,46]. Furthermore, the study population consisted solely of patients of Romanian descent, which may limit the generalizability of the findings to other ethnic populations. Additionally, we did not assess the impact of these coagulation abnormalities on clinical outcomes such as bleeding, transfusion requirements, or mortality. The results of this study open new perspectives for future research, including the long-term follow-up of patients to assess outcomes such as mortality and major adverse cardiovascular events (MACE).

## Conclusions

Our results suggest that patients undergoing CABG with additional intraoperative interventions experience more severe derangements in coagulation and hemostasis parameters, along with a higher degree of liver dysfunction at discharge compared to admission, as quantified by the MELD score. These findings have important implications for the perioperative management of this high-risk patient population and warrant further investigation to elucidate the mechanisms underlying these observations and their impact on clinical outcomes. The association with the MELD score highlights the necessity of closely monitoring liver function and coagulation profiles in these patients. Additionally, it indicates that factors beyond the MELD score, such as the patient's overall health status and vulnerability, should be carefully considered when evaluating their postoperative risk.

## References

[1] Ohri SK, Benedetto U, Luthra S (2022). Coronary artery bypass surgery in the UK, trends in activity and outcomes from a 15-year complete national series. Eur J Cardiothorac Surg.

[2] Hannan EL, Racz M, Culliford AT (2013). Risk score for predicting in-hospital/30-day mortality for patients undergoing valve and valve/coronary artery bypass graft surgery. Ann Thorac Surg.

[3] Hirata N, Sawa Y, Matsuda H (1999). Predictive value of preoperative serum cholinesterase concentration in patients with liver dysfunction undergoing cardiac surgery. J Card Surg.

[4] Klemperer JD, Ko W, Krieger KH (1998). Cardiac operations in patients with cirrhosis. Ann Thorac Surg.

[5] Giallourakis CC, Rosenberg PM, Friedman LS (2002). The liver in heart failure. Clin Liver Dis.

[6] Alvarez AM, Mukherjee D (2011). Liver abnormalities in cardiac diseases and heart failure. Int J Angiol.

[7] Pandey CK, Karna ST, Pandey VK, Tandon M, Singhal A, Mangla V (2012). Perioperative risk factors in patients with liver disease undergoing non-hepatic surgery. World J Gastrointest Surg.

[8] Søreide JA, Deshpande R (2021). Post hepatectomy liver failure (PHLF) - recent advances in prevention and clinical management. Eur J Surg Oncol.

[9] Modi A, Vohra HA, Barlow CW (2010). Do patients with liver cirrhosis undergoing cardiac surgery have acceptable outcomes?. Interact Cardiovasc Thorac Surg.

[10] An Y, Xiao YB, Zhong QJ (2007). Open-heart surgery in patients with liver cirrhosis: indications, risk factors, and clinical outcomes. Eur Surg Res.

[11] El Amir M, Gamal Eldeen H, Mogawer S (2015). Different score systems to predict mortality in living donor liver transplantation: which is the winner? The experience of an Egyptian center for living donor liver transplantation. Transplant Proc.

[12] Vanhuyse F, Maureira P, Mattei MF, Laurent N, Folliguet T, Villemot JP (2013). Use of the model for end-stage liver disease score for guiding clinical decision-making in the selection of patients for emergency cardiac transplantation. Eur J Cardiothorac Surg.

[13] Wang ZX, Yan LN, Wang WT, Xu MQ, Yang JY (2007). Impact of pretransplant MELD score on posttransplant outcome in orthotopic liver transplantation for patients with acute-on-chronic hepatitis B liver failure. Transplant Proc.

[14] McPhail MJ, Farne H, Senvar N, Wendon JA, Bernal W (2016). Ability of King’s College Criteria and model for end-stage liver disease scores to predict mortality of patients with acute liver failure: a meta-analysis. Clin Gastroenterol Hepatol.

[15] Färber G, Marx J, Scherag A, Saqer I, Diab M, Sponholz C, Doenst T (2023). Risk stratification for isolated tricuspid valve surgery assisted using the model for end-stage liver disease score. J Thorac Cardiovasc Surg.

[16] O'Leary JG, Friedman LS (2007). Predicting surgical risk in patients with cirrhosis: from art to science. Gastroenterology.

[17] Peters SA, Colantonio LD, Dai Y (2021). Trends in recurrent coronary heart disease after myocardial infarction among us women and men between 2008 and 2017. Circulation.

[18] De Vecchis R, Di Biase G, Esposito C (2013). Statin use for nonrheumatic calcific aortic valve stenosis: a review with meta-analysis. J Cardiovasc Med (Hagerstown).

[19] Diodato M, Chedrawy EG (2014). Coronary artery bypass graft surgery: the past, present, and future of myocardial revascularisation. Surg Res Pract.

[20] Jones ML, Qiu S, Sudarshan C (2010). Perioperative outcomes in hybrid versus conventional surgical coronary artery revascularisation. Interact Cardiovasc Thorac Surg.

[21] Frolov A, Lobov A, Kabilov M (2023). Multi-omics profiling of human endothelial cells from the coronary artery and internal thoracic artery reveals molecular but not functional heterogeneity. Int J Mol Sci.

[22] Watanabe S, Arayawudhikul N (2022). Minimally invasive direct coronary artery bypass to the left anterior descending artery using right gastroepiploic artery graft for a redo case with poor conduits. Indian J Thorac Cardiovasc Surg.

[23] Nalysnyk L, Fahrbach K, Reynolds MW, Zhao SZ, Ross S (2003). Adverse events in coronary artery bypass graft (CABG) trials: a systematic review and analysis. Heart.

[24] Scrutinio D, Giannuzzi P (2008). Comorbidity in patients undergoing coronary artery bypass graft surgery: impact on outcome and implications for cardiac rehabilitation. Eur J Cardiovasc Prev Rehabil.

[25] Tran L, Williams-Spence J, Shardey GC, Smith JA, Reid CM (2019). The Australian and New Zealand Society of Cardiac and Thoracic Surgeons database program - two decades of quality assurance data. Heart Lung Circ.

[26] Nashef SA, Roques F, Sharples LD, Nilsson J, Smith C, Goldstone AR, Lockowandt U (2012). EuroSCORE II. Eur J Cardiothorac Surg.

[27] Rus M, Ardelean AI, Judea Pusta C (2023). Prevalence of cardiovascular comorbidities in patients with rheumatoid arthritis. Medicina (Kaunas).

[28] Pathare P, Elbayomi M, Weyand M, Griesbach C, Harig F (2023). MELD-score for risk stratification in cardiac surgery. Heart Vessels.

[29] Yu DC, Chen WB, Jiang CP, Ding YT (2013). Risk assessment in patients undergoing liver resection. Hepatobiliary Pancreat Dis Int.

[30] Bollano E, Redfors B, Rawshani A (2022). Temporal trends in characteristics and outcome of heart failure patients with and without significant coronary artery disease. ESC Heart Fail.

[31] Tandon R, Froghi S (2021). Artificial liver support systems. J Gastroenterol Hepatol.

[32] Salis S, Mazzanti VV, Merli G, Salvi L, Tedesco CC, Veglia F, Sisillo E (2008). Cardiopulmonary bypass duration is an independent predictor of morbidity and mortality after cardiac surgery. J Cardiothorac Vasc Anesth.

[33] Howard RJ, Crain C, Franzini DA, Hood CI, Hugli TE (1988). Effects of cardiopulmonary bypass on pulmonary leukostasis and complement activation. Arch Surg.

[34] Nemeth N, Furka I, Miko I (2014). Hemorheological changes in ischemia-reperfusion: an overview on our experimental surgical data. Clin Hemorheol Microcirc.

[35] Ades PA, Keteyian SJ, Wright JS (2017). Increasing cardiac rehabilitation participation from 20% to 70%: a road map from the Million Hearts cardiac rehabilitation collaborative. Mayo Clin Proc.

[36] Nelson M, Green J, Spiess B, Kasirajan V, Nicolato P, Liu H, Meshkin RS (2018). Measurement of blood loss in cardiac surgery: still too much. Ann Thorac Surg.

[37] Mirzaei S, Hershberger PE, DeVon HA (2019). Association between adverse clinical outcomes after coronary artery bypass grafting and perioperative blood transfusions. Crit Care Nurse.

[38] Lamelas J (2017). Minimal access tricuspid valve surgery. Ann Cardiothorac Surg.

[39] Kapur NK, Davila CD, Jumean MF (2017). Integrating interventional cardiology and heart failure management for cardiogenic shock. Interv Cardiol Clin.

[40] Burkauskas J, Lang P, Bunevičius A, Neverauskas J, Bučiūtė-Jankauskienė M, Mickuvienė N (2018). Cognitive function in patients with coronary artery disease: a literature review. J Int Med Res.

[41] Im GY, Lubezky N, Facciuto ME, Schiano TD (2014). Surgery in patients with portal hypertension: a preoperative checklist and strategies for attenuating risk. Clin Liver Dis.

[42] Kaltenbach MG, Mahmud N (2023). Assessing the risk of surgery in patients with cirrhosis. Hepatol Commun.

[43] Northup PG, Wanamaker RC, Lee VD, Adams RB, Berg CL (2005). Model for End-Stage Liver Disease (MELD) predicts nontransplant surgical mortality in patients with cirrhosis. Ann Surg.

[44] De Martin E, Sapisochin G (2020). Predicting liver transplant patient outcomes. Is a validated model enough?. Transplantation.

[45] Saugel B, Fletcher N, Gan TJ, Grocott MP, Myles PS, Sessler DI (2024). PeriOperative Quality Initiative (POQI) international consensus statement on perioperative arterial pressure management. Br J Anaesth.

[46] Story DA (2008). Postoperative complications in elderly patients and their significance for long-term prognosis. Curr Opin Anaesthesiol.

